# Single Photon Computed Tomography-Myocardial Perfusion Scintigraphy. Diagnostic Tool Anticipating the Disease

**DOI:** 10.5935/abc.20180265

**Published:** 2019-02

**Authors:** Whady Hueb

**Affiliations:** Instituto do Coração (InCor) Hospital das Clínicas da Faculdade de Medicina da Universidade de São Paulo (FMUSP), São Paulo, SP - Brazil

**Keywords:** Obesity, Diabetes Mellitus, Myocardial Reperfusion/radionuclide imaging, Coronary Artery Disease/physiopathology

Without making any value judgment, a large percentage of physicians who request tests to make diagnoses is observed in the contemporary clinical practice. On the other hand, a considerable percentage of physicians who make the diagnosis and possibly ask for tests to confirm the diagnosis is also observed. Both behaviors are considered valid when common good is achieved: The patients’ benefit. However, the request for tests without an appropriate criterion is not only harmful to the patient, but also to the system.

In present-day medicine, there is a large collection of tests considered normal throughout the medical knowledge area, including cardiology. In a publication by Dippe Jr et al.^1^ in this issue, the authors, in a retrospective analysis of a database, found this trend. Of 5,526 scans of myocardial perfusion scans performed on obese patients (grade 1), 77% were considered normal. Assuming that the exams were requested for investigation of myocardial ischemia, the authors related the presence of perfusion deficit with myocardial ischemia in only 23%. Based on these data, they found, after applying a "creative statistic", a 245% risk-ratio for typical angina. It is known, in principle, that perfusion deficit is an expression of intrinsic myocardial abnormality.

The coronary arterial system may or may not contribute to this anomaly. In this scenario, the disproportion between supply and consumption of O_2_ by the myocardium has some variables that do not always depend on the coronary arterial system. Thus, to relate the presence of perfusion deficit as a future prediction of clinical variables may be a methodologically dangerous path. Still in this study, the authors mentioned obesity as a predictor of diabetes mellitus in 57% of the people studied. In this case, the subjects had grade 1 obesity at the upper limit of the Body Mass Index (BMI) for overweight.

On the other hand, this classification imposes limitations to its application.

There are some problems with using BMI as a determinant of obesity. Muscular people have high BMIs and are not obese. The elderly need a differentiated classification to determine obesity. Moreover, the World Health Organization concluded that Asian people could be considered obese even with a BMI of 25. Thus, unless better judged, the levels of obesity reported in this research cannot be disseminated as a predictor of the prognosis. Up to a point, this study resembles the study model by Hachamovitch et al.^2^ where the authors, in methodologically biased studies and results subject to discussion, established a percentage of myocardial ischemia close to 12% as a reference for indication of myocardial revascularization. Unfortunately, these results are referenced in the main specialty guidelines. Abstaining from practicing prediction, these results should be placed in the collections of transient truths.
